# Impact of anger emotional stress before pregnancy on adult male offspring

**DOI:** 10.18632/oncotarget.22007

**Published:** 2017-10-24

**Authors:** Sheng Wei, Xinyang Xiao, Jieqiong Wang, Shiguang Sun, Zifa Li, Kaiyong Xu, Fang Li, Jie Gao, Dehao Zhu, Mingqi Qiao

**Affiliations:** ^1^ Department of Neurosurgery, Qilu Hospital of Shandong University, Brain Science Research Institute, Shandong University, Jinan 250012, China; ^2^ Lab of Traditional Chinese Medicine Classical Theory, Ministry of Education, Shandong University of Traditional Chinese Medicine, Jinan 250355, China; ^3^ Lab of Behavioral Brain Analysis, Shandong University of Traditional Chinese Medicine, Jinan 250355, China; ^4^ Second Affiliated Hospital of Shandong University of Traditional Chinese Medicine, Shandong Provincial Hospital of Integrated Medicine, Jinan 250001, China; ^5^ Fengtai Maternal and Children's Health Hospital of Beijing, Beijing 100069, China

**Keywords:** stress before pregnancy, anger emotion, aggressive behavior test, resident intruder paradigm, monoamine neurotransmitters

## Abstract

Previous studies have reported that maternal chronic stress or depression is linked to an increased risk of affective disorders in progeny. However, the impact of maternal chronic stress before pregnancy on the progeny of animal models is unknown. We investigated the behaviors and the neurobiology of 60-day-old male offspring of female rats subjected to 21 days of resident-intruder stress before pregnancy. An anger stressed parental rat model was established using the resident-intruder paradigm and it was evaluated using behavioral tests. Anger stressed maternal rats showed a significant increase in locomotion and aggression but a reduction in sucrose preference. Offspring subjected to pre-gestational anger stress displayed enhanced aggressive behaviors, reduced anxiety, and sucrose preference. Further, offspring subjected to pre-gestational stress showed significant impairments in the recognition index (RI) on the object recognition test and the number of platform crossings in the Morris water maze test. The monoaminergic system was significantly altered in pre-gestationally stressed offspring, and the expression of phosphorylated cyclic adenosine monophosphate response element binding protein (P-CREB), brain-derived neurotrophic factor (BDNF), and serotonin transporter (SERT) levels in pre-gestational stressed offspring were altered in some brain regions. Fluoxetine was used to treat pre-gestational stressed maternal rats and it significantly reduced the changes caused by stress, as evidenced by both behaviors and neural biochemical indexes in the offspring in some but not all cases. These findings suggest that anger stress before pregnancy could induce aggressive behaviors, cognitive deficits, and neurobiological alterations in offspring.

## INTRODUCTION

Previous studies have shown that maternal stress has a negative influence on the behaviors of offspring, and it is related to frequently occurring affective diseases among them [1–3]. Epidemiological evidence has indicated that negative spiritual and physiological stresses during pregnancy are risk factors for a series of adverse pregnancy phenomena, including premature delivery and low infant birth weight [4]. These findings were also supported by a few other studies [5–7]. Studies have also indicated that maternal stress during pregnancy can predict cognitive ability and fearfulness in infancy [8].

Most animal studies concerning the influence of maternal stress on offspring have concentrated on its effects before delivery [9–13], with few examining the influence of pre-gestational maternal stress upon offspring. It has recently been reported that the male offspring of dams exposed to chronic unpredictable stress (CUS) before pregnancy are at high risk of developing depressive-like behaviors, and that the abnormal expression of phosphorylated cyclic adenosine monophosphate response element binding protein (P-CREB), brain-derived neurotrophic factor (BDNF), and N-methyl-D-aspartate receptor (NMDA-R) subunits in the hippocampus might play a role in the mechanisms underlying these depressive-like behaviors in CUS offspring [14, 15]. Researchers have previously used the CUS paradigm [14, 15] to examine the influence of a variety of physical stressors such as electric shock or restraint.

Compared to depression, anger is an extreme negative emotional response that significantly influences interpersonal relationships and social harmony. It is also the most stressful reaction and is associated with pathogenesis. As the core symptom of anger, aggressive behavior has been used as a measure of anger across extant literature. The substrate of aggressive action is a physiological cascade that involves γ-aminobutyric acid (GABA) and serotonin (5-HT), as well as neurosteroids [16–18]. This results in the over-activation of the amygdaloid nucleus, which is in charge of impulse control, in turn causing the prefrontal cortex to malfunction [19–24]. Hormones also play an important role in human aggression. Moderate levels of progesterone, high levels of testosterone, and lowered levels of corticosterone appear to mediate aggression [25–29]. Furthermore, allosteric modification of GABAAR is closely related to aggressive behavior. Moderate levels of allopregnanolone, high levels of androstenediol, and a positive endogenous modifier of GABAAR can increase aggressiveness [30–33]. Decreased 5-HT can also lead to aggressive behavior and decreased expression of 5-HT1A receptors in the prefrontal cortex, and increased raphe nucleus density are relevant to aggression [34].

It is uncertain how maternal anger stress before pregnancy influences the offspring and what molecular mechanism and signal transduction pathways are involved. Hence, this study proposed to induce anger stress among maternal rats in social isolation, combined with the resident-intruder mode, before conducting drug intervention with Fluoxetine. This would enable us to observe the influence of anger stress in maternal rats before pregnancy on the behavioral and neurobiological indexes of adult offspring, which in turn would help explain the role of heredity in the transfer of maternal anger to the offspring and the microscopic mechanism therein, from the perspective of embryology.

## RESULTS

### Resident-intruder induced maternal anger stress model preparation and behavioral evaluation

**Figure 1:**
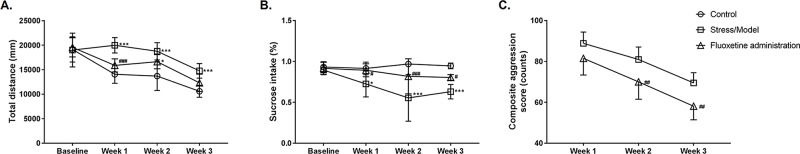
Maternal behavioral detection for the control group (Control), anger stress (Stress/Model) and Fluoxetine treated (Fluoxetine administration) groups **(A)** The total distance of rat moving in 6 min was measured and recorded as the open-field test score (n=8). **(B)** The percentage of sucrose solution consumed in total liquid consumption was calculated as sucrose preference in the experiment (n=8). **(C)** The composite aggression score was calculated and used to evaluate the degree of aggression (n = 8). ^*^: P<0.05 versus the control; ^***^: P<0.001 versus the control; ^#^: P<0.05 versus the model; ^##^: P<0.01 versus the model; ^###^: P<0.001 versus the model.

From the 1st to 3rd week of modeling, the subsequent Bonferroni post hoc test revealed that the scores of the model group in the open field test increased significantly as compared to that of the control group (Figure 1A, P<0.0001, P<0.0001, P=0.0003, respectively). Fluoxetine effectively lowered the scores on the open field test, but this effect was significant only for the 1st week (Figure 1A, P=0.0004). A two-way analysis of variance (ANOVA) revealed a significant effect of stress [F(2, 84)=26.78, P<0.0001] and time [F(3, 84)=42.97, P<0.0001].

Similar results were obtained for the sucrose preference experiment. Subsequent Bonferroni post hoc tests revealed that, before modeling, i.e., at baseline, there was no statistical difference in the sucrose preference percentage for the rats in all groups. From the 1st to 3rd week of modeling, the sucrose preference percentage for the rats in the model group significantly decreased as compared to that of the control group (Figure 1B, P=0.0255, P<0.0001, P<0.0001, respectively). Fluoxetine can effectively decrease the sucrose preference percentage caused by the modeling, and we observed a significant decrease in sucrose preference in our study (Figure 1B, P=0.0366, P=0.0004, P=0.0277, respectively). A two-way ANOVA revealed a significant effect of stress [F(2, 76)=24.88, P<0.0001] and time [F(3, 76)=4.711, P=0.0045].

Since no resident-intruder experiment was conducted on the rats in the control group, a composite aggression value could not be obtained, and the aggressive behaviors of the model and control groups could not be compared. Subsequent Bonferroni post hoc tests revealed that there was a significant difference in the value of composite aggression from the 2nd to 3rd week between the model and Fluoxetine administered groups (Figure 1C, P=0.0076, P=0.0051, respectively), i.e., Fluoxetine effectively lowered the value of composite aggression. A two-way ANOVA revealed a significant effect of stress [F(1, 42)=25.37, P<0.0001] and time [F(2, 42)=39.7, P<0.0001].

### Adult male offspring emotional behaviors and learning memory detection

**Figure 2:**
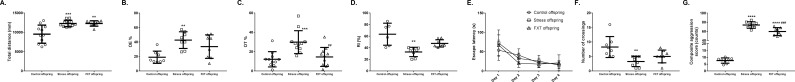
Offspring behavioral detection for the control (Control offspring), anger stress (Stress offspring) and Fluoxetine treated (FXT offspring) groups **(A)** The total distance of the adult offspring rat moving in 6 min was measured and recorded as the open-field test score (n=11 for both Control offspring and Stress offspring; n=8 for FXT offspring). **(B)** The percentage of open-arms entries was indicated as OE % in the elevated-plus maze test (n=8). **(C)** The percentage of time for offspring in open-arms was indicated as OT % in the elevated-plus maze test (n=11). **(D)** In the object recognition task, the recognition index (RI) was calculated to measure novel object recognition and it was used as the main index for retention (n=6 for Control offspring; n=8 for both Stress offspring and FXT offspring). **(E)** Escape latency in the Morris water maze test was recorded during Day 4 place-learning and it was used as a measure of acquisition and retrieval of the spatial information necessary to reach the platform location (n=10). **(F)** On the fifth day of the Morris water maze test, the number of platform crossings was recorded to determine the degree of learning acquired by the rats with respect to the position of the platform in the pool (n=8). **(G)** The composite aggression score was calculated for the offspring to evaluate the degree of aggression (n=8). ^**^: P<0.01 versus the control; ^***^: P<0.001 versus the control; ^****^: P<0.0001 versus the control; ^##^: P<0.01 versus the model; ^###^: P<0.001 versus the model.

The scores (total distance) of the offspring on the open field test differed significantly among groups [F(2, 27)=10.98, P=0.0003]. Subsequent Bonferroni post hoc tests revealed that the scores of the adult male offspring in the model and Fluoxetine administration groups were significantly higher as compared to those of the control offspring (Figure 2A, 12273±831.4 vs. 9522±2343, P=0.0009; 12287±691.6 vs. 9522±2343, P=0.0021, respectively).

In the EPM experiment, the percentages of open-arms entries (OE %) and time/duration in open-arms (OT %) of adult offspring differed significantly across groups [F(2, 21)=7.334, P=0.0038; F(2, 30)=10.19, P=0.0004, respectively]. Subsequent Bonferroni post hoc tests revealed that the OE % of the model group offspring significantly increased as compared to that of the control group offspring (Figure 2B, 42.64±11.55 vs. 18.95±8.431, P=0.0032). Additionally, adult offspring in the Fluoxetine administration group showed a similar tendency as compared to the normal control group, but the difference was nonsignificant (Figure 2B, 33.67±16.24 vs. 18.95±8.431, P=0.0846). Bonferroni post hoc tests also revealed that the OT % of the model group offspring significantly increased as compared to that of the control offspring (Figure 2C, 29.82±11.85 vs. 12.07±7.927, P=0.0007). In contrast, as compared with the model offspring, the percentage of open-arms entries of adult Fluoxetine administration offspring decreased significantly (Figure 2C, 14.5±9.827 vs. 29.82±11.85, P=0.0035).

In the ORT experiment, the recognition index (RI percentage) of adult offspring differed significantly across groups [F(2, 19)=12.12, P=0.0004]. Subsequent Bonferroni post hoc tests revealed that the RI percentage of the model offspring was significantly lower as compared to that of the control offspring (Figure 2D, 32.75±8.061 vs. 63.37±18.99, P=0.0003). Similarly, the RI percentage of the Fluoxetine administration group was lower than that of the control offspring, but this difference was nonsignificant (Figure 2D, 47.54±6.268 vs. 63.37±18.99, P=0.0601). Further, as compared to the RI percentage of the model offspring, that of the Fluoxetine administration offspring was higher, but this difference was not significant (Figure 2D, 47.54±6.268 vs. 32.75±8.061, P=0.0571).

With reference to the Morris water maze test, a two-way ANOVA revealed a significant effect of time (time effect) in the escape latency of adult offspring among the groups [F(3, 105)=37.91, P<0.0001] during the training period. Specifically, subsequent Bonferroni post hoc tests revealed no significant differences among the three groups during the first four days of training (Figure 2E, P>0.05). However, the number of platform crossings measured on the fifth day revealed a significant difference among groups [F(2, 21)=7.357, P=0.0038]. Subsequent Bonferroni post hoc tests revealed that, as compared to the control offspring, the number of platform crossings of the model offspring was significantly lower (Figure 2F, 3.25±1.753 vs. 8.25±3.615, P=0.0033). Further, as compared to the model offspring, the number of platform crossings of the Fluoxetine administration group was higher, but this difference was not significant (Figure 2F, 5±2.204 vs. 3.25±1.753, P=0.6003).

The aggressive behavior test results showed that the composite aggression scores of the offspring differed significantly across groups [F(2, 21)=227.9, P<0.0001]. Subsequent Bonferroni post hoc tests revealed that, as compared to the control offspring, the aggressive behavior score was higher in the offspring in model and Fluoxetine administration groups (Figure 2G, 73.65±7.102 vs. 7.613±1.949, P<0.0001; 59.42±8.543 vs. 7.613±1.949, P<0.0001, respectively). Further, the aggressive behavior score of the Fluoxetine administration offspring was significantly lower than that of the model offspring (Figure 2G, 59.42±8.543 vs. 73.65±7.102, P=0.0008).

### Offspring abnormal behavior phenotype and 5-HT related neurobiological relations

**Figure 3:**
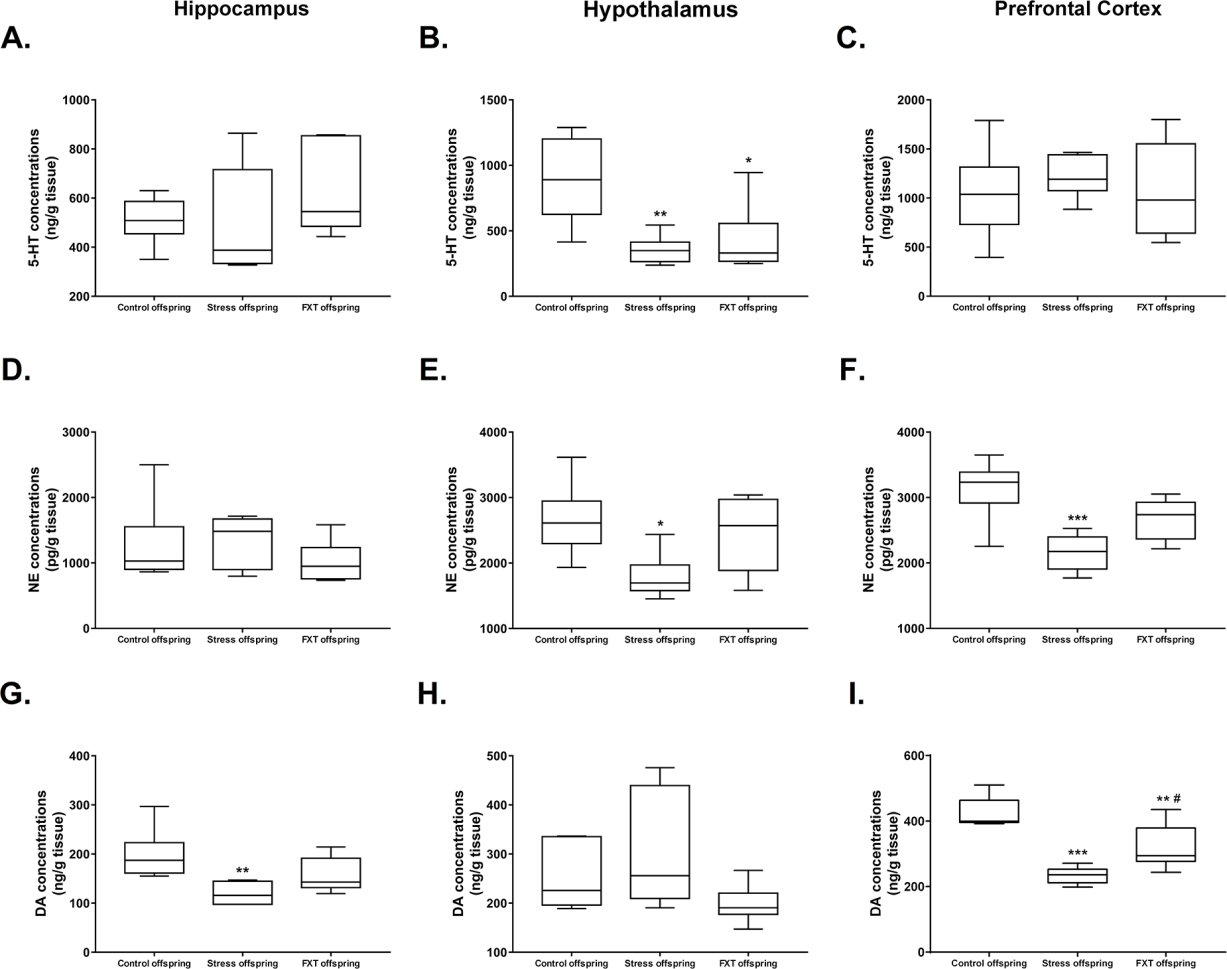
**(A–C)** 5-HT levels in the hippocampus (A), hypothalamus (B), and prefrontal cortex (C) of the offspring of each group. **(D–F)** NE levels in the hippocampus (D), hypothalamus (E), and prefrontal cortex (F) of the offspring of each group. **(G–I)** DA levels in the hippocampus (G), hypothalamus (H), and prefrontal cortex (I) of the offspring of each group. FXT offspring: adult male offspring of rats from the Fluoxetine administration group. ^*^: P<0.05 versus the control; ^**^: P<0.01 versus the control; ^***^: P<0.001 versus the control; ^#^: P<0.05 versus the model. n=6 for all.

The 5-HT content in the left hippocampus and left prefrontal cortex of the offspring in the three groups did not differ significantly [F(2, 14)=1.14, P=0.3479; F(2, 15)=0.3038, P=0.7424, respectively]. Subsequent Bonferroni post hoc tests revealed no statistically significance differences in the pairwise comparison (Figure 3A and 3C, for 5-HT content in the left hippocampus: 510±94.01 vs. 495±221.6 vs. 644.6±194.9 for the control, model and Fluoxetine administration groups, respectively; for 5-HT content in the left prefrontal cortex: 1044 ± 454.1 vs. 1217 ± 212.4 vs. 1075 ± 504.2 for the control, model and Fluoxetine administration groups, respectively). However, 5-HT content in the hypothalamus exhibited significant differences among groups [F(2, 15)=8.252, P=0.0038]. Subsequent Bonferroni post hoc tests revealed that the 5-HT content in the hypothalamus of the model and Fluoxetine administration offspring was significantly lower as compared to that of the control offspring (Figure 3B, 354.3±106.6 vs. 892.3±326, P=0.0059; 426.7 ± 261.5 vs. 892.3 ± 326, P=0.0165, respectively).

Among offspring in all groups, there was no significant difference in the NE content in the left hippocampus [F(2, 15)=0.8175, P=0.4603]. Subsequent Bonferroni post hoc tests revealed no statistically significant differences in pairwise comparisons (Figure 3D, 1263±621.9 vs. 1346±389.4 vs. 1018±321.9 from the control, model, and Fluoxetine administration offspring, respectively). However, the NE content in left hypothalamus and left prefrontal cortex exhibited significant difference among groups [F(2, 15)=4.917, P=0.0228; F(2, 15)=10.86, P=0.0012, respectively]. Subsequent Bonferroni post hoc tests revealed that for the NE content in the hypothalamus as well as the left prefrontal cortex was significantly lower in the model offspring as compared to that of the control offspring (Figure 3E, 1787±342.6 vs. 2653±550.9, P=0.0271; Figure 3F, 2159±279.8 vs. 3135±467.5, P=0.0009, respectively).

There was no significant difference in the hypothalamus DA content across the three groups [F(2, 15)=2.505, P=0.1152]. Pairwise comparisons in the subsequent Bonferroni post hoc tests revealed no statistically significant differences (Figure 3H, 251.9±67.41 vs. 303.6±118.3 vs. 197.9±38.96 for the control, model, and Fluoxetine administration groups, respectively). However, the DA content in the left hippocampus and left prefrontal cortex exhibited significant differences among the three groups [F(2, 15)=6.319, P=0.0102; F(2, 15)=22, P<0.0001, respectively]. Subsequent Bonferroni post hoc tests revealed that the DA content in left hippocampus of the model offspring was significantly lower as compared to that of the control offspring (Figure 3G, 119.7±22.85 vs. 198.2±51.13, P=0.0087). Further, as compared with the control offspring, the DA content in left prefrontal cortex of the model and Fluoxetine administration offspring was significantly lower (Figure 3I, 234.2±25.16 vs. 424.3±46.66, P<0.0001; 319.4±67.9 vs. 424.3±46.66, P=0.0071, respectively). Finally, in comparison to the model offspring, the DA content in left prefrontal cortex of the Fluoxetine administration group was significantly higher (Figure 3I, 319.4±67.9 vs. 234.2±25.16, P=0.0287).

**Figure 4:**
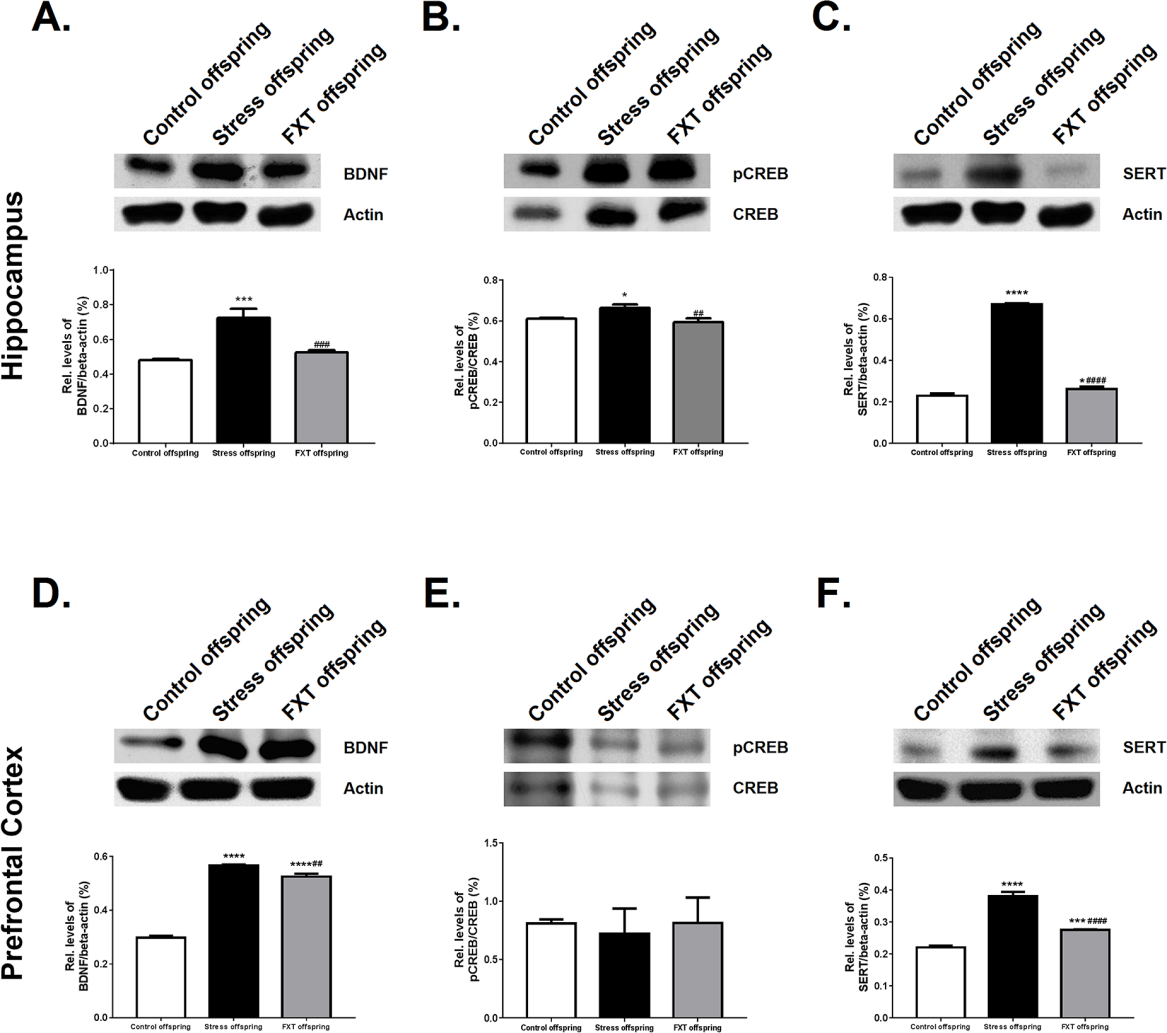
Relative levels of BDNF/β-actin in the hippocampus **(A)** and prefrontal cortex **(D)** of the offspring of each group. Relative levels of pCREB/CREB in the hippocampus **(B)** and prefrontal cortex **(E)**. Relative levels of SERT/β-actin in the hippocampus **(C)** and prefrontal cortex **(F)**. FXT offspring: adult male offspring of rats from the Fluoxetine administration group. ^*^: P<0.05 versus the control; ^***^: P<0.001 versus the control; ^****^: P<0.0001 versus the control; ^##^: P<0.01 versus the model; ^###^: P<0.001 versus the model; ^####^: P<0.0001 versus the model. n=3 for all.

The Western blot was adapted to detect BDNF, P-CREB and 5-HTT protein expression in the right hippocampus and right prefrontal cortex of the offspring in all three groups. In the right hippocampus, the BDNF, P-CREB, and 5-HTT protein expressions were significantly different among the offspring of the three groups [F(2, 6)=49.18, P=0.0002; F(2, 6)=15.86, P=0.0040; F(2, 6)=1695, P<0.0001, respectively]. Subsequent Bonferroni post hoc tests revealed that the BDNF, P-CREB, and 5-HTT protein expressions in the model offspring were significantly higher as compared with those of the control offspring (Figure 4A-4C, 0.7228±0.05347 vs. 0.4784±0.009545, P=0.0003; 0.6618±0.01771 vs. 0.6108±0.004653, P=0.0205; 0.6688±0.006157 vs. 0.2293±0.01145, P<0.0001, respectively). In the right hippocampus, the BDNF, P-CREB, 5-HTT protein expressions in the Fluoxetine administered offspring were significantly higher as compared to those of the model offspring (Figure 4A-4C, 0.5255±0.01127 vs. 0.7228±0.05347, P=0.0008; 0.5933±0.01955 vs. 0.6618±0.01771, P=0.0049; 0.2614±0.01223 vs. 0.6688±0.006157, P<0.0001, respectively).

In addition, in the right prefrontal cortex, the BDNF and 5-HTT expressions of the offspring differed significantly across the three groups [F(2, 6)=1083, P<0.0001; F(2, 6)=285.4, P<0.0001]. Subsequent Bonferroni post hoc tests revealed that the BDNF and 5-HTT expressions of the model offspring were significantly higher as compared to those of the control offspring (Figure 4D and 4F, 0.567±0.003001 vs. 0.298±0.006881, P<0.0001; 0.3812±0.01319 vs. 0.2204±0.005681, P<0.0001, respectively). Further, their expressions in the Fluoxetine administration offspring were significantly lower as compared with those of the model offspring (Figure 4D and 4F, 0.5251±0.01085 vs. 0.567±0.003001, P=0.0016; 0.2748±0.002197 vs. 0.3812±0.01319, P<0.0001, respectively). The P-CREB level in right prefrontal cortex of the offspring did not differ significantly across groups [F(2, 6)=0.2664, P=0.7747]. Pairwise comparisons in the Bonferroni post hoc tests revealed that the control offspring, model offspring and Fluoxetine administration group offspring exhibited no statistically significant differences (Figure 4E, 0.8101±0.03451 vs. 0.7197±0.218 vs. 0.8138±0.2175, respectively).

## DISCUSSION

### Anger stress rat model

Numerous preliminary studies have attempted to duplicate the emotional pathogenic animal model, but as emotions are random and complex, it is difficult to know how mental and physical stress can be differentiated in stress studies. In previous research on the anger stress model, processes like electric shocks or restraints were commonly adopted to induce stress [14, 15]. However, these stress stimuli were unavoidably mixed with physical stress elements. This could change the physiological status of animals and ultimately influence their behavioral reactions, which in turn would not sufficiently elucidate the reactive patterns of mental and physiological stress [35].

The animal model adopted in this study utilized the methods used by Megane [36], Albonetti [37], and Miczek [38], according to which experiment rats tend to be aggressive because of stress after long-term isolated feeding, they instinctively initiate self-defense against intruders, and they demonstrate an angry mindset and behavior. This kind of “resident-intruder” model reduced physical stress to an almost negligible degree; hence, it was a type of angry animal model closer to human angry mood that was mainly caused by mental stress elements. Further, we combined an aggressive behavior test with an open field test and a sucrose preference test to evaluate the behavioral changes of rats after anger stress. Meanwhile, the selective 5-HT re-uptake inhibitor Fluoxetine [39] was adopted to treat the model, after discovering that Fluoxetine could effectively reduce the behavioral changes caused by the modeling. This was done to ultimately verify the success of the anger stress model.

The resident-intruder paradigm could also be induced as a depression model, i.e., the social defeat model [40]. According to this model, resident rats attack the intruder because of territory consciousness and behavior. However, if intruders were larger in terms of body weight and physical reserve, with richer experience for battle, resident rats would always be defeated by the emotional experience, which could mimic the pathological changes in clinical depression. However, the opposite occurred in our study, as the resident rats always won, which enhanced their emotional aggressiveness. The comparison of aggressive behaviors between the model group and Fluoxetine administration group confirmed this point, although there was no control group in this analysis.

The -parental model revealed mania, irritability, and aggression after the 21-day resident-intruder stress, which was significantly reduced by Fluoxetine administration (Figure 1C). This finding was consist with previously reported works [41, 42]. Aggressive behavior, considered as the core symptom of anger, is believed to be triggered by anger-provoking stimuli [43]. Hence, our results indicated that resident-intruder stress could induce anger emotional experiences in the modeled rats, and that such experience was relieved by Fluoxetine. The total distance in OFT increased after stress (Figure 1A), reflecting that the model rats’ locomotor and exploratory activity increased. Other clinical and animal tests have also reported similar findings [42, 44], indicating that anger emotional experience is expressed as more dynamic, excited, and stimulated. Normally, rats become more active after experiencing resident-intruder stress, which is the winners’ behavior and is different from that of the losers [45]. Our SPT results revealed that animals showed a general decline in sucrose preference (Figure 1B) after stress, which is indicative of anhedonia [46]. It was probably because resident-intruder stress specifically reduced the desire to experience pleasure.

### Behavior evaluations of model offspring

In current work, only male offspring were studied for two reasons: (i) to avoid interference of hormone on the behavior of the offspring; (ii) the series of behavioral experiments seemed to be too many for female offspring to survive. Female offspring exhibited weaker tolerance and died more easily than male offspring did. However, the experimental design employed in the present study was based on common practice [14, 47]. Animal behavior in an open field test is a kind of comprehensive behavior that includes fear of the strange environment, high degree of stimulation, and continuous exploration [48]. In this study, total distance was used to measure the anger behaviors of the model offspring, and a greater distance indicated higher anger. However, behavioral changes could also be explained with reference to the enhanced activity and exploration ability of the model offspring as well as the increased exploration duration of the strange environment, since the scores of the adult male model offspring were significantly higher as compared with those of the control offspring (P=0.0009; Figure 2A).

The elevated plus maze test offered contradictory mental choices to rats, reflecting both their explorative nature in strange environments and their fearful mentality about open arms during uphang. Our experiment index used the open-arms entry (OE) percentage and open-arms time (OT) percentage to reflect the anxiety of the animal; the OE % and OT % of anxious animals obviously decreased, and the anti-anxiety drugs increased both [49]. The present results showed that the OE % and OT % of the model offspring were significantly higher than those of the control offspring were (Figure 2B and 2C, P=0.0032, P=0.0007), indicating that the model offspring were less anxious. However, it is difficult to explain this finding. Though the resident-intruder condition induced anger stress, which induced anhedonia, as evident from the decreased sucrose preference (Figure 1B), anger stress also led the rats to exhibit higher total distance in the OFT (Figure 1A), which is contrary to the belief that social defeat stress induces depressive-like behaviors. Further, as evidenced by the offspring's behaviors, the consequences of anger stress were different from that of social defeat stress (Figure 2A-2C). These differences in both parents and offspring could provide evidence for the need to distinguish resident-intruder stress and social defeat stress. Actually, this difference between the behavioral profiles of anxiety-like behavior and depressive-like behavior is being reported in more studies [50–52]. We noted that the total number of times model offspring exhibited open- and close-arms entries was significantly higher than that of the control offspring (data not shown), indicating an enhanced activity level in model offspring, which was consistent with the conclusion in the open field test (in which total distance hinted enhanced activity).

Object recognition tests aim to assess animal learning memory. They are based on the rationale that animals have innate tendencies to explore new objects [53]. The results of our study showed that the RI of model offspring was significantly lower as compared to that of the control offspring (Figure 2D, P=0.0003), indicating that model offspring showed no preference over new objects and formed no effective memory of old objects. This not only showed that the learning ability of model offspring was influenced, but also that memory storage and extraction was weakened, suggesting that anger stress led to defects in non-spatial learning memory abilities among rats. The offspring in the Fluoxetine administration group exhibited a higher RI as compared to the model offspring, but there was no significant difference (Figure 2D, P=0.0601). This may indicate that Fluoxetine capsules could reverse the adverse effects of anger stress on learning memory, but the effect was not statistically significant.

The classic Morris water maze test reflects the ability to develop spatial reference memory, and the storage and extraction ability of long time interval spatial reference memory because the information processing mechanism evoked in this test involves limbic systems like the hippocampus, corpus striatum, forebrain, and cerebral cortex-related encephalic regions [54–56]. The number of platform crossings of model offspring was significantly lower than that of the control offspring (Figure 2F, P=0.0033), and the strategy seeking mode of model offspring was marginal and random, indicating that the spatial reference memory storage and extraction ability of model offspring was damaged.

In the aggressive behavior test, control offspring exhibited normal behaviors, i.e., they did not show intensive aggressive behaviors when intruding rats entered the cage. The behaviors of the offspring in model group and Fluoxetine administration group were different. When intruding rats entered the cage of a resident rat, the latter quickly switched into aggressive status by attacking and biting from the front and sides. Intruding rats were always in a disadvantageous position with the high frequency of on-top attacks that they were subjected to, and sometimes, they slipped into a “stupor” state. Through analyzing the aggressive behavior test video and data statistics, the adult offspring in model and Fluoxetine administration groups showed mania, irritability, and aggressiveness, and their composite aggressive behavior score was significantly higher than that of the control offspring (Figure 2G, P<0.0001, P<0.0001, respectively). This finding indicates that the maternal anger stress modeling may have induced the offspring to show the same anger. Further, a comparison of the model and Fluoxetine administration offspring revealed that Fluoxetine had remarkable treatment effects on the offspring anger induced by maternal anger before pregnancy (Figure 2G, P=0.0008). This result suggested that Fluoxetine was not only able to correct behaviors induced by anger mood in the parental generation, but that it also cured aggressive behaviors in the offspring that were caused by parental stress.

### Monoaminergic system and transcriptional regulation factors

Under normal situations, the secretion of 5-HT, NE, and DA is maintained at certain level to maintain the stability of neuroregulation function [57]. Our results indicated that the level of these three monoamine neurotransmitters in encephalic regions of model offspring underwent changes (Figure 3). These changes could be related to a defect in the learning and memory ability [58, 59]. A drop in the level of monoamine neurotransmitters in the hypothalamus could result in the hyperfunction of the hypothalamic pituitary adrenocortical (HPA) axis, leading to an increase in adrenocorticotrophic hormone and corticosterone levels. On the other hand, the expression of glucocorticoid receptors (GRs) in the hippocampus increased. An increase in corticosterone levels could harm GRs by binding them and further inducing dysfunction of the hippocampus, which is related to learning and memory ability [60, 61]. Results of the ORT and MWM tests in the present study provide indirect evidence for the above prediction.

This study indicated that the offspring of rats who got pregnant soon after receiving resident-intruder anger stress would exhibit increased BDNF expression in the right hippocampus and right prefrontal cortex (Figure 4A and 4D, P=0.0003, P<0.0001). Increased BDNF expression would influence the synaptic plasticity establishment of offspring and would ultimately damage their cognitive function [62]. However, we fail to reasonably explain how pregestational anger stress induces BDNF expression changes in the offspring, which is worthy of further research.

As the third messenger in cells, CREB can regulate the genetic transcription and protein expression of various neuropeptides and neurotrophic factors [63], influence the survival and growth of neurons and synaptic plasticity, affect the formation of long-term memory, and regulate the excitability of the neural system [57, 64–67]. The present results show that changes in P-CREB and BDNF protein expressions in the offspring were consistent (Figure 4B and 4E), indicating that the cAMP-CREB-BDNF signal path may play an important role in anger, leading to pathogenesis and cognitive function damage.

It has been widely reported that dysfunction in serotonergic neurotransmission is involved in various mental disorders, including depression [68] and anxiety [69]. Therefore, changes in the expression and function of SERT are considered closely related to the pathophysiology of these disorders. The current study showed that the 5-HTT levels of the model group offspring were significantly higher as compared to those of the control offspring (Figure 4C and 4F, P<0.0001, P<0.0001, respectively). These findings are in line with the results of praxiology experiments. However, it is still not clear how maternal anger stress before pregnancy induces these neurological function changes in the offspring and how it influences their behaviors.

Few reports have examined the influence of the pregestational anger stress model on the behaviors and cognitive ability of adult male offspring. In the current work, we induced parental aggressive behaviors using the resident-intruder paradigm before pregnancy and observed the changes in emotional behavior and learning memory, monoamine neurotransmitter content, and levels of key proteins in the signaling pathway. Our results confirmed that the impact of pregestational anger stress could be transmitted across generations. We additionally attempted to interpret offspring behavioral changes and cognitive ability damage based on neurobiological indexes, even though these changes seemingly failed to obtain a consistent interpretative conclusion. Based on the findings on biological indexes, we conclude that there was no significant difference among groups or that the drug intervention did not lead to any obvious correction of the abnormal fluctuations in the biological indexes. We predict that the manifestation of anger mood observed in the present study was not completely consistent with clinical syndromes because it had not reached the stage of disease; therefore, the treatment effect of the drug intervention for the abnormal fluctuations in some biological indexes may not have been evident. According to reported evidences, we assumed the maternal stress before pregnancy could cause an imbalance in the nervous-endocrine-immune network, which may have further resulted in hormonal abnormalities in the fetus’ HPA axis through the mother-placenta-fetus surface [70]. These abnormalities in neurosteroids could influence the SERT level via glucocorticoid receptors, and it could influence the 5-HT, NE, and DA contents. These neurotransmitters function as the first messenger and, through CREB activation, they generated depression-like or anxiety-like behaviors [71]. However, we did not evaluate hormone changes in the HPA axis of both mother and offspring, which could be one of the limitations of the current work. Hence, it is important to examine if the above assumption is correct.

The current study provides evidence that aggressive behaviors induced by the resident-intruder paradigm before pregnancy could result in a negative impact on adult male offspring. An interesting challenge is that it was unclear if aggressive behaviors seemed to be induced by the resident-intruder paradigm, while the depression-like behaviors seemed to be induced by the social-defeat stress. Our current work, particularly the observations of offspring behavior, presented some evidence of the ethology to distinguish these behaviors. However, to identify the differences in the consequences of both types of stress on offspring, we should have induced both behaviors before pregnancy.

## MATERIALS AND METHODS

### Ethics statement

Animal experiments were performed in accordance with the Guide for the Care and Use of Laboratory Animals, formulated by the National Institute of Health, USA, and were approved by the Institutional Committee for Animal Care and Use of Shandong University of Traditional Chinese Medicine (Approval ID: DWSY201404013).

### Animals and grouping

Forty-five female specific pathogen-free (SPF) Wistar rats, aged 6 weeks, weighing 120–150 g, 20 female SD Wistar rats (intruder) weighing 110–130 g, and 20 male SPF Wistar rats (used for mating) weighing 120–150 g were purchased from Beijing Vital River Experimental Animal Technology Co. Ltd. (SCXK [JING] 2007-0001) (Beijing, China). The animals were housed under a reversed 12/12 h light/dark cycle (lights off 08:00). They had ad libitum access to food and water, except for during experiments. The animals were habituated to the maintenance conditions for 1 week and were handled daily (control phase). The room temperature was maintained at 22±1°C with 50% humidity and freely available food and water. Rats were minimally handled and soiled bedding was periodically replaced only partially, without removing the rats, such that the home-cage odors, nests, etc., were minimally disrupted [72]. The intruder rats were housed in a separate room (one rat per cage) and they subjected to conditions identical to those described above.

All rat subjects were tested. Of these, 36 that had similar sucrose preference test (SPT) and open field test (OFT) scores were selected. These selected rats were randomly divided into the following three groups before beginning of stressful procedures: control group (n=12), anger stress group (n=12) and Fluoxetine administration group (n =12). After grouping, the anger stress and Fluoxetine administration groups were bred in isolation (one rat per cage), while the control group was continued to be raised socially (five per cage).

### Drugs

Fluoxetine was purchased from Eli Lilly Co. (Indianapolis, IN; approval no. H20090463) and was administered intragastrically (2.7 mg/Kg/d) to the animals at 08:30 every morning, as per the requirements of the experiment.

### Resident-intruder anger emotional stress procedure

**Figure 5:**
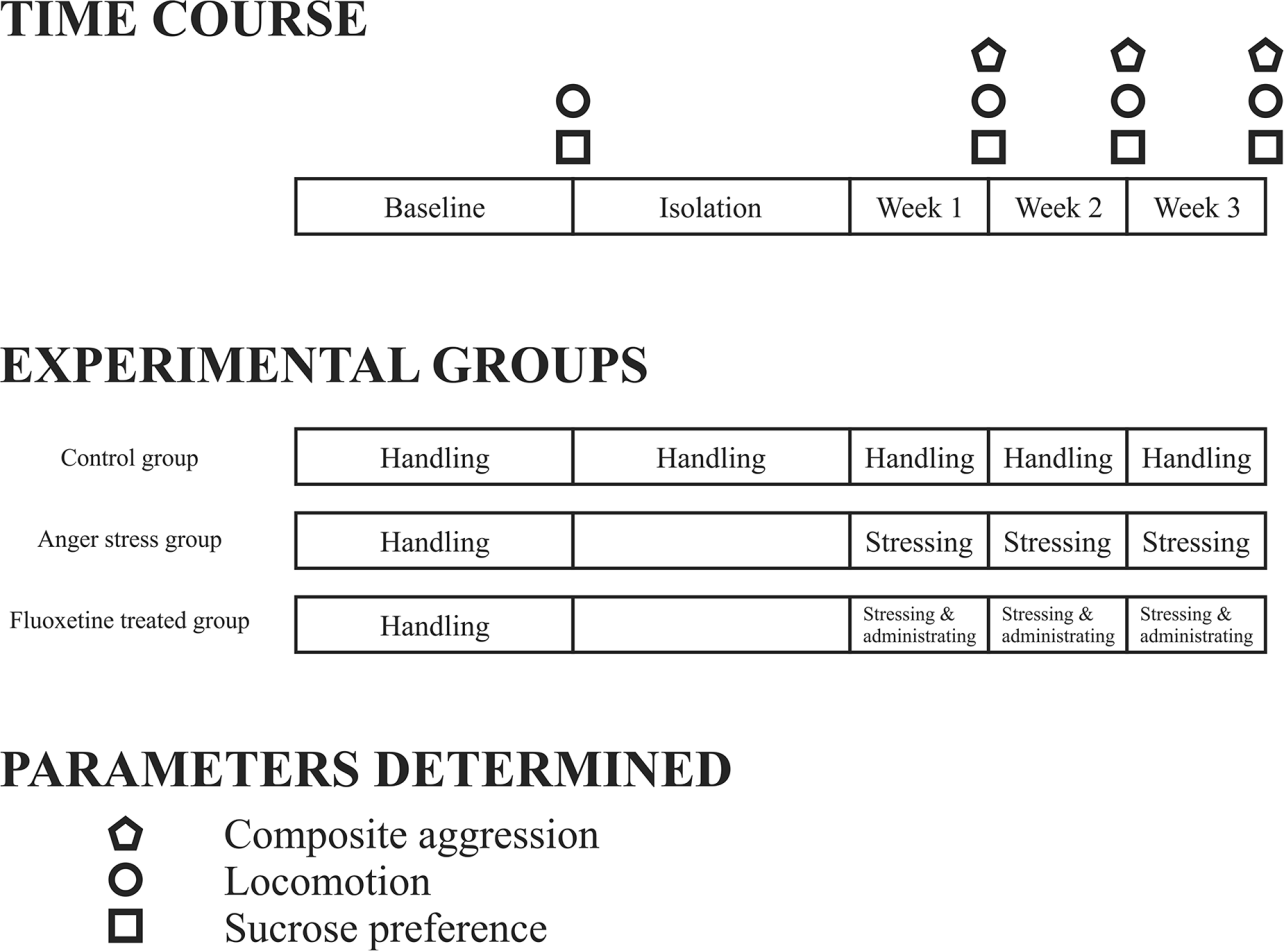
Time course of maternal anger stressing and behavioral detection in the control, anger stress, and Fluoxetine treated groups

The maternal resident-intruder procedure and behavioral analysis process has been presented in Figure 5. One week after adapting to the environment, each resident rat was housed in a separate cage in a quiet environment to build the social isolation model. This was followed by the resident-intruder experiment one week later. This experiment was performed in accordance with our previous study [73], in which the resident-intruder experiment was conducted at 12:00 noon daily, because reversed day and night rats were in an excitatory state within during this period. SD intruding rats were placed in the cage of the resident rats for 15 min. The first five minutes before timing were considered as the adaptive period, and from the 6th to 15th minutes we assessed aggressive behaviors, observed the behaviors of the resident rats, and recorded the behavioral changes of the rats between 6 to 15 min. The resident-intruder experiment was conducted once daily for a period of 3 weeks on the resident rats. The intruding rats brought into each test were screened by the Latin square design [74], to ensure that they displayed normal aggressive behaviors.

The behavioral expressions of resident rats were recorded and analyzed daily, including number of attacks made, attack duration, number of on-top attacks, on-top duration, number of bites, and piloerection. The aggressive behavior test was video recorded every week for a blind behavioral evaluation. The record was played back by three persons who had undergone the same training to evaluate the aggressive behaviors of rats, with a consistency Kappa>0.95. Composite aggression was calculated using the following formula: Composite aggression=[(number of attacks)+0.2×(attack duration)+(number of bites)+0.2×(on-top duration)+(piloerection)].

### Open field test (OFT)

Automatic recording of open-field activity was performed using the XR-SuperMaze animal behavior video tracking and analysis system (Shanghai Xinruan Information Technology Co. Ltd, Shanghai, China), which was equipped with a standard, open, plexiglas arena (100 cm×100 cm×50 cm). Each animal was placed in the center of the experimental apparatus immediately prior to testing, and were allowed to explore it for 6 min. The arena was carefully cleaned with ethyl alcohol (70%) after every test. A computer was used to record the trajectory of the animals, and the following behaviors were analyzed: total distance moved, distance travelled in the center and peripheral areas, time spent in the center area [75, 76], and number of rears.

### Sucrose preference test (SPT)

During the entire experiment, SPTs were performed at weekly intervals (starting from baseline). Two bottles of liquid were provided to rats ad libitum, over a 24 h period. One bottle contained a 0.8% sucrose solution, and the other bottle contained tap water. To prevent the possible effects of side preference on the results, bottle positions were switched after 12 h. Rats were provided ad libitum access to food and water before the experiment since food and water deprivation before the test was unnecessary. Sucrose preference was calculated from the amount of sucrose solution consumed, which was expressed as the percentage of the total amount of liquid consumed [77].

### Offspring of the dams

Seven days after the maternal resident-intruder procedure was completed, female rats were housed in pairs for mating with sexually experienced males of the same strain (ratio 2:1). The day on which sperm was observed in vaginal smears was designated as embryonic day 0 (E 0). Nesting material was provided to each gestational dam, which were singly housed and not disturbed. The day of delivery was designated as postnatal day 0 (PND 0). The pups were weaned at 22 days of age, and then housed in groups of three to four rats based on their sex. All subsequent experiments described below were performed on the male offspring at 2 months of age. Twelve pups from each group (control, anger stress and Fluoxetine administration groups) were used (n=12), with one or two pups from each mother.

### Elevated-plus maze (EPM) test

The EPM was made of black Perspex, with arms 50 cm long and 10 cm wide. The dimensions of center zone were 10 cm length×10 cm width. The apparatus was elevated to a height of 50 cm above the floor. The test room was illuminated by red dim light (about 30 lux). The closed arms were surrounded by a 40 cm wall, the open ones presented 0.5 cm edges in order to maximize open-arm entries [78]. Rats were placed in the center of a cross maze, facing an open arm [79, 80] and were allowed to explore the maze for 5 minutes. After each test, the EPM was cleaned with ethyl alcohol (70%) to remove any scent cues left by the preceding subject. Two paws had to be inside the line indicating the entrance to an arm to signal the start of the time spent in a specific arm. The time spent in the different arms of the maze was recorded. The number of entries into the various arms of the maze were also determined and served as a measure of the locomotor activity of the rat.

### Object recognition task (ORT)

Male offspring were tested over four consecutive days in the open field arena, according to the previously described object recognition task protocol [81, 82]. The test room was illuminated by white bright light (about 200 lux). On Day 1 and 2, 5-min habituation sessions were performed at 10:00 a.m. in order to let the animals freely explore the arena. The open field apparatus consisted of a square box, 40 cm wide, 40 cm long, and 40 cm high. On Day 3, twenty-four hours after the last habituation session, rats were subjected to a 5-min training session where they were presented with two identical, non-toxic objects (i.e., two metal cans) that were placed against a wall of the open field arena. To prevent coercion to explore the objects, each rat was released in the center of the opposite wall with its back to the objects. The time spent exploring each object was recorded using the SuperMaze Video Tracking System (Shanghai Xinruan Information Technology Co. Ltd, Shanghai, China). A 2-cm^2^ area surrounding the object was defined such that nose entries were recorded as the time spent exploring the object. After the training session, animals were placed in their home cage for a 1 h retention interval. Subsequently, animals were returned to the arena containing two objects: one was identical to the familiar object but was previously unused (to prevent olfactory cues and the necessity to wash objects during experimentation), while the other was a novel object (metal, glass, or hard plastic item). The time spent exploring each object was recorded over a 5-min period. On Day 4, after the 24 h retention period, rats were tested again using two objects, i.e., the familiar object and another novel object. Objects were randomized and counterbalanced across animals. Again, the time spent exploring each object was recorded over a 5-min period. The objects and arena were thoroughly cleaned with 70% ethyl alcohol at the end of each experimental session.

The recognition index (RI), which is the time spent investigating the novel object divided by the total amount of time spent exploring both the novel and familiar objects [RI = TN/(TN + TF)], is relative to the total time spent exploring objects. It is a measure of novel object recognition and the main index used for retention. An RI percentage greater than 50% indicates more time spent exploring the novel object, whereas that less than 50% indicates that the time was prevailingly spent exploring the familiar object. An RI of 50% indicates a null preference.

### Morris water maze (MWM) test

The MWM apparatus used in these experiments consisted of a circular, light-blue swimming pool with the following dimensions: diameter, 160 cm; wall height, 70 cm. It was filled with tap water to a depth of 50 cm. The water temperature was carefully maintained at 23±2°C. The pool was divided into four quadrants (North-West [NW], North-East [NE], South-West [SW], and South-East [SE]) of equal size using the SuperMaze system to quadrisect the pool. A removable squared escape platform (10 cm×10 cm) could be positioned in the quadrants, with the center 30 cm away from the wall and 1.5 cm below the level of the water, in order to be invisible to the swimming rat. The pool was placed in an experimental room lit by white bright light (about 200 lux) that had several extra-maze cues (e.g., bookshelves and posters), and was not moved in the room throughout the entire experimental period. An automatic video tracking system (SuperMaze Video Tracking System, Shanghai, China) was used to record animal movements in the pool. This software provided us with measures of escape latency, path length and the time spent in each quadrant.

The animals were submitted to the following training protocol according to a protocol described by Plescia et al. [81]. Place learning with multiple trials (days 1–4): place learning consisted of training the rats to escape from the water by reaching a hidden platform placed in the SE zone where it was maintained throughout the experimental session. Rats were placed into the pool facing the walls of each quadrant, in the following starting point order: SW, NW, NE, and SE. Each animal underwent four trials per day over four consecutive days, and they were allowed to swim until the escape platform was found (escape latency), for a maximum of 120 s. When the platform was reached, they were left on it for 15 s. If rats did not find the escape platform within 120 s, the experimenter guided them gently to the platform where they were allowed to remain for 15 s to reinforce the information from the visuo-spatial cues in the environment. Animals were returned to their home cages and briefly warmed under a heating lamp during the 5 min inter-trial intervals. The parameters recorded were as follows: escape latency (s) as a measure of acquisition and retrieval of the spatial information necessary to reach the platform location, and path length (m) as an additional element in search strategies. Trial duration, reinforcement time on the platform, and all other experimental conditions were as before.

Probe (day 5): After the animal had completed the 4-day place-learning task, it was placed in the pool for 120 s on the fifth day without the goal platform, and the number of platform crossings was recorded. The transfer test is used to determine the degree of learning the animals have acquired with respect to the position of the platform in the pool.

### Tissue and blood collection

After the behavior tests were completed, the rats were executed. They were decapitated to obtain 6 ml blood, which was then subject to 20 min centrifugation in a 3000-rpm centrifugal machine, and the serum was be stored in a -80°C cryogenic refrigerator separately. The whole brain, including the hypothalamus, hippocampus, and prefrontal cortex, was stripped on an ice block; the contents of 5-HT, NE, DA of the hypothalamus, left hippocampus, and left prefrontal cortex were tested using a kit; and BDNF, P-CREB, 5-HTT protein expressions of the right hippocampus and right prefrontal cortex were tested through a Western blot analysis.

### Monoamine neurotransmitter concentration analyses

NE, 5-HT, and DA concentrations in the hypothalamus, hippocampus, and prefrontal cortex were measured in 50–100 μl of brain tissue samples using individual commercially available enzyme immunoassay kits (CUSABIO^®^, China), as previously described [79].

### Western blotting

Western blotting was performed according to standard procedures [83]. Antibodies against BDNF (Abcam, Cambridge, MA), SERT (Abcam), CREB (Cell Signaling Technology, Danvers, MA), pCREB (Cell Signaling Technology), and β-actin (Sigma-Aldrich, St. Louis, MO) were used to detect target proteins at dilution ratios recommended by the instruction manual. A Goat anti-Rabbit IgG-HRP (Abmart, Berkeley Heights, NJ) was used as the secondary antibody at a dilution of 1:2000. Immuno-positive bands were visualized using a chemiluminescent method (G:BOX chemiXR5, SYNGEN, Sacramento, CA). The optical densities of the target protein bands visible on the X-ray film were determined densitometrically using Gel-Pro32 software [84]. All Western blotting experiments were repeated at least three times.

### Statistical analysis

Data were analyzed using Graph Pad Prism version 6.0.1 (GraphPad Software, Inc., San Diego, CA). Outliers were defined as two or more standard deviations from the mean and were removed from the analysis [72]. The data were tested for normality (Kolmogorov-Smirnov test) and homoscedasticity (Levene's test) before being analyzed using either unpaired t-tests or parametric repeated measures ANOVA. Behavioral data were analyzed using unpaired t-tests or two-way ANOVA. Neurochemical and biochemical data were analyzed using unpaired t-tests. For all analyses, post hoc comparisons were conducted after ANOVAs where appropriate using a Bonferroni protected least significance test. Data were presented as means±standard deviation. A P-value less than 0.05 was considered statistically significant for differences determined via the ANOVA.

## Abbreviations

CUS: unpredictable stress
P-CREB: phosphorylated cyclic adenosine monophosphate response element binding protein
BDNF: brain-derived neurotrophic factor
SERT/5-HTT: serotonin transporter/5-HT transporter
GABA: γ-aminobutyric acid
5-HT: serotonin
OE: open-arms entry
OT: open-arms time
SPF: specific pathogen-free
OFT: open Field Test
SPT: sucrose Preference Test
PND 0: postnatal day 0
EPM: elevated-Plus Maze
ORT: object Recognition Task
RI: recognition index
DA: dopamine
NE: norepinephrine
SD: standard deviation
